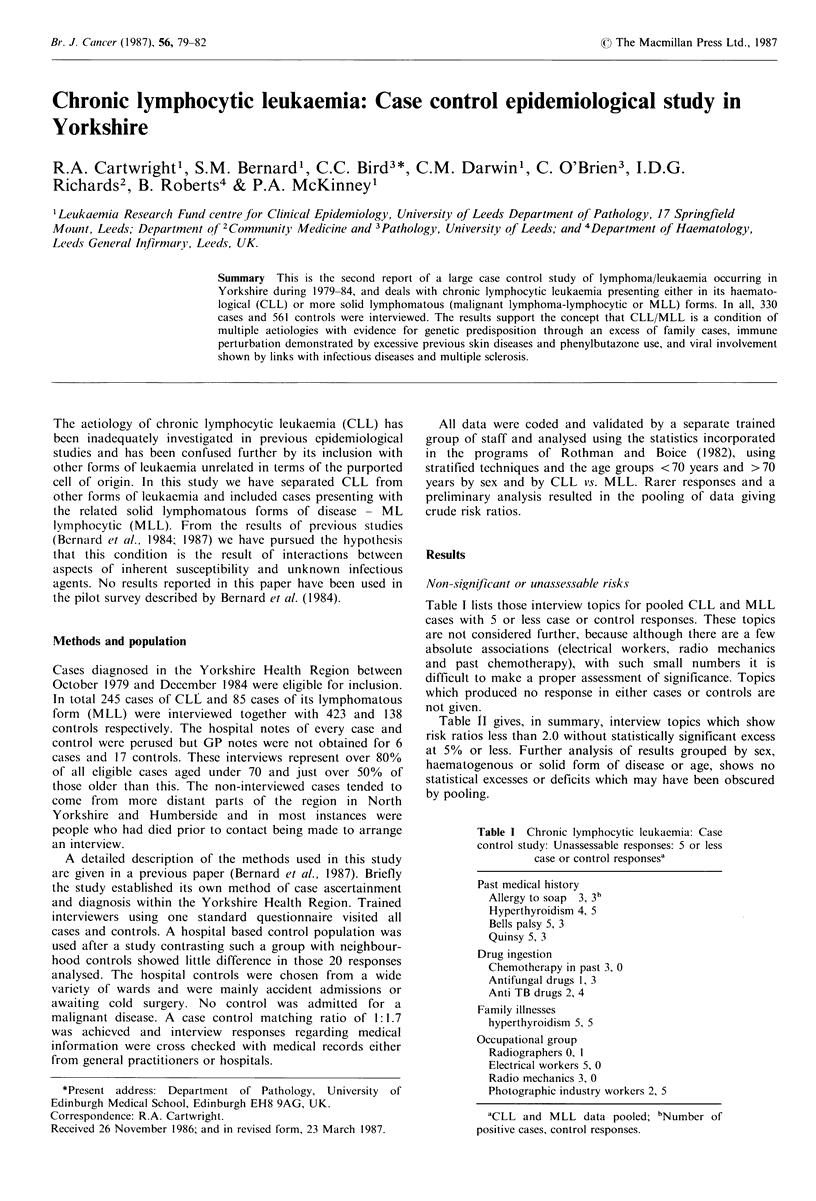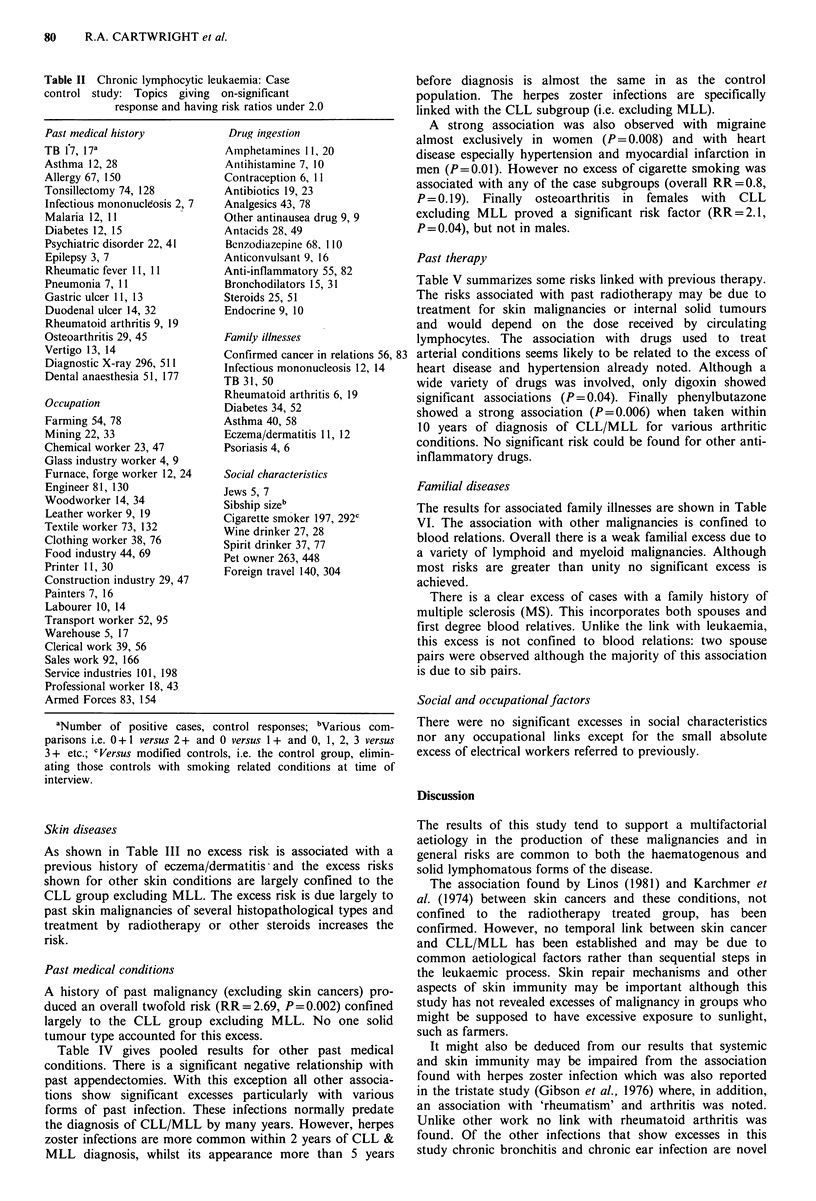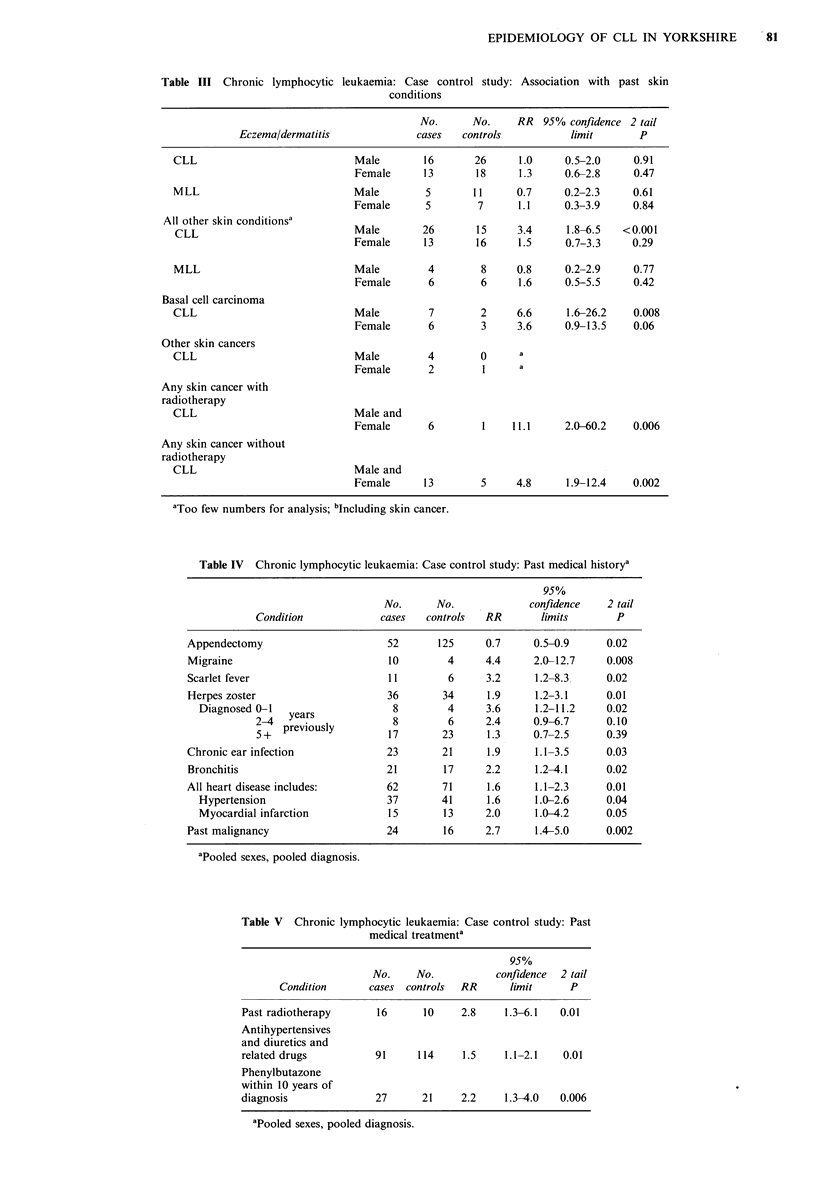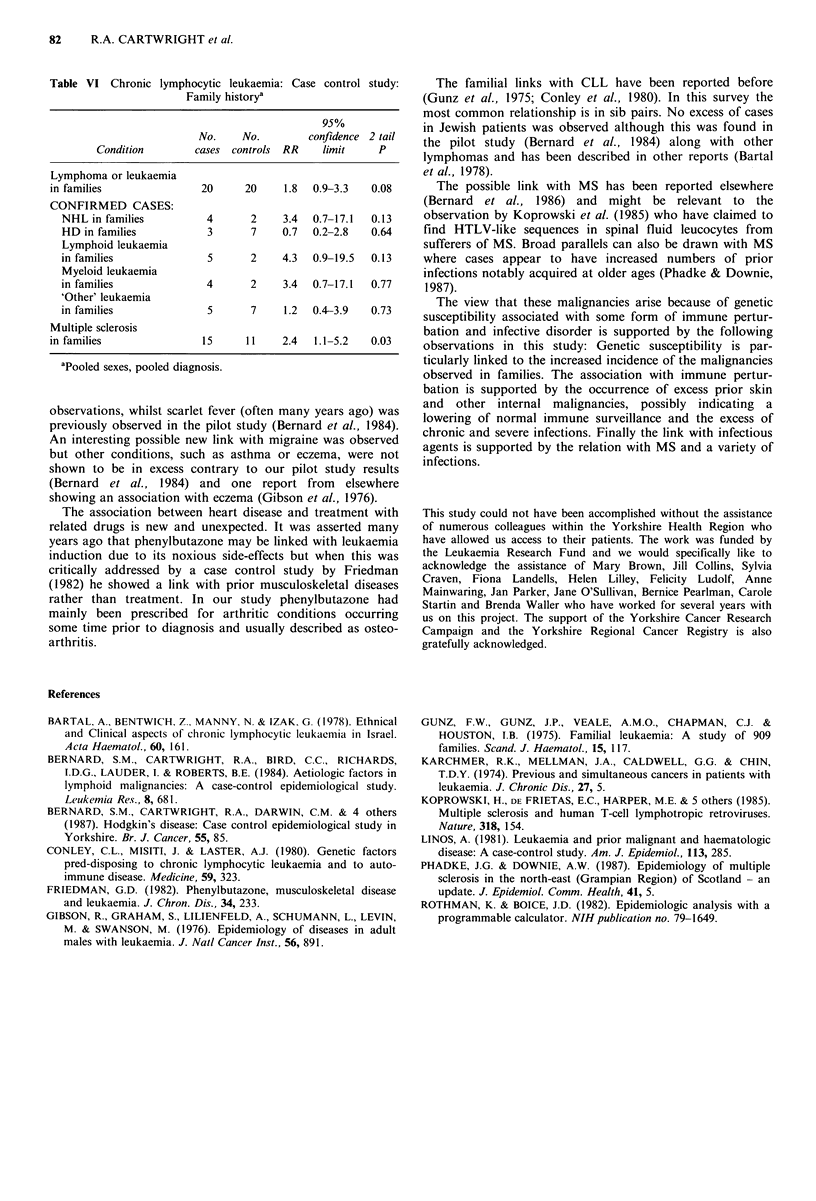# Chronic lymphocytic leukaemia: case control epidemiological study in Yorkshire.

**DOI:** 10.1038/bjc.1987.158

**Published:** 1987-07

**Authors:** R. A. Cartwright, S. M. Bernard, C. C. Bird, C. M. Darwin, C. O'Brien, I. D. Richards, B. Roberts, P. A. McKinney

## Abstract

This is the second report of a large case control study of lymphoma/leukaemia occurring in Yorkshire during 1979-84, and deals with chronic lymphocytic leukaemia presenting either in its haematological (CLL) or more solid lymphomatous (malignant lymphoma-lymphocytic or MLL) forms. In all, 330 cases and 561 controls were interviewed. The results support the concept that CLL/MLL is a condition of multiple aetiologies with evidence for genetic predisposition through an excess of family cases, immune perturbation demonstrated by excessive previous skin diseases and phenylbutazone use, and viral involvement shown by links with infectious diseases and multiple sclerosis.


*Br. J. Cancer* (1987), **56**, 79–82 © The Macmillan Press Ltd., 1987

### Chronic lymphocytic leukaemia: Case control epidemiological study in Yorkshire

R.A. Cartwright[1], S.M. Bernard[1], C.C. Bird[3]*, C.M. Darwin[1], C. O'Brien[3], I.D.G. Richards[2], B. Roberts[4] & P.A. McKinney[1]

[1]*Leukaemia Research Fund centre for Clinical Epidemiology, University of Leeds Department of Pathology, 17 Springfield Mount, Leeds; Department of* [2]*Community Medicine and* [3]*Pathology, University of Leeds; and* [4]*Department of Haematology, Leeds General Infirmary, Leeds, UK.*

**Summary** This is the second report of a large case control study of lymphoma/leukaemia occurring in Yorkshire during 1979–84, and deals with chronic lymphocytic leukaemia presenting either in its haematological (CLL) or more solid lymphomatous (malignant lymphoma-lymphocytic or MLL) forms. In all, 330 cases and 561 controls were interviewed. The results support the concept that CLL/MLL is a condition of multiple aetiologies with evidence for genetic predisposition through an excess of family cases, immune perturbation demonstrated by excessive previous skin diseases and phenylbutazone use, and viral involvement shown by links with infectious diseases and multiple sclerosis.

The aetiology of chronic lymphocytic leukaemia (CLL) has been inadequately investigated in previous epidemiological studies and has been confused further by its inclusion with other forms of leukaemia unrelated in terms of the purported cell of origin. In this study we have separated CLL from other forms of leukaemia and included cases presenting with the related solid lymphomatous forms of disease – ML lymphocytic (MLL). From the results of previous studies (Bernard *et al.*, 1984; 1987) we have pursued the hypothesis that this condition is the result of interactions between aspects of inherent susceptibility and unknown infectious agents. No results reported in this paper have been used in the pilot survey described by Bernard *et al.* (1984).

### Methods and population

Cases diagnosed in the Yorkshire Health Region between October 1979 and December 1984 were eligible for inclusion. In total 245 cases of CLL and 85 cases of its lymphomatous form (MLL) were interviewed together with 423 and 138 controls respectively. The hospital notes of every case and control were perused but GP notes were not obtained for 6 cases and 17 controls. These interviews represent over 80% of all eligible cases aged under 70 and just over 50% of those older than this. The non-interviewed cases tended to come from more distant parts of the region in North Yorkshire and Humberside and in most instances were people who had died prior to contact being made to arrange an interview.

A detailed description of the methods used in this study are given in a previous paper (Bernard *et al.*, 1987). Briefly the study established its own method of case ascertainment and diagnosis within the Yorkshire Health Region. Trained interviewers using one standard questionnaire visited all cases and controls. A hospital based control population was used after a study contrasting such a group with neighbourhood controls showed little difference in those 20 responses analysed. The hospital controls were chosen from a wide variety of wards and were mainly accident admissions or awaiting cold surgery. No control was admitted for a malignant disease. A case control matching ratio of 1:1.7 was achieved and interview responses regarding medical information were cross checked with medical records either from general practitioners or hospitals.

*Present address: Department of Pathology, University of Edinburgh Medical School, Edinburgh EH8 9AG, UK.
Correspondence: R.A. Cartwright.
Received 26 November 1986; and in revised form, 23 March 1987.

All data were coded and validated by a separate trained group of staff and analysed using the statistics incorporated in the programs of Rothman and Boice (1982), using stratified techniques and the age groups <70 years and >70 years by sex and by CLL *vs.* MLL. Rarer responses and a preliminary analysis resulted in the pooling of data giving crude risk ratios.

### Results

### *Non-significant or unassessable risks*

Table I lists those interview topics for pooled CLL and MLL cases with 5 or less case or control responses. These topics are not considered further, because although there are a few absolute associations (electrical workers, radio mechanics and past chemotherapy), with such small numbers it is difficult to make a proper assessment of significance. Topics which produced no response in either cases or controls are not given.

Table II gives, in summary, interview topics which show risk ratios less than 2.0 without statistically significant excess at 5% or less. Further analysis of results grouped by sex, haematogenous or solid form of disease or age, shows no statistical excesses or deficits which may have been obscured by pooling.

**Table I** Chronic lymphocytic leukaemia: Case control study: Unassessable responses: 5 or less case or control responses[a]

| |
|---|
| Past medical history |
| Allergy to soap 3, 3[b] |
| Hyperthyroidism 4, 5 |
| Bells palsy 5, 3 |
| Quinsy 5, 3 |
| Drug ingestion |
| Chemotherapy in past 3, 0 |
| Antifungal drugs 1, 3 |
| Anti TB drugs 2, 4 |
| Family illnesses |
| hyperthyroidism 5, 5 |
| Occupational group |
| Radiographers 0, 1 |
| Electrical workers 5, 0 |
| Radio mechanics 3, 0 |
| Photographic industry workers 2, 5 |

[a]CLL and MLL data pooled; [b]Number of positive cases, control responses.

**Table II** Chronic lymphocytic leukaemia: Case control study: Topics giving on-significant response and having risk ratios under 2.0

*Past medical history*

TB 17, 17[a]

Asthma 12, 28

Allergy 67, 150

Tonsillectomy 74, 128

Infectious mononucleosis 2, 7

Malaria 12, 11

Diabetes 12, 15

Psychiatric disorder 22, 41

Epilepsy 3, 7

Rheumatic fever 11, 11

Pneumonia 7, 11

Gastric ulcer 11, 13

Duodenal ulcer 14, 32

Rheumatoid arthritis 9, 19

Osteoarthritis 29, 45

Vertigo 13, 14

Diagnostic X-ray 296, 511

Dental anaesthesia 51, 177

*Occupation*

Farming 54, 78

Mining 22, 33

Chemical worker 23, 47

Glass industry worker 4, 9

Furnace, forge worker 12, 24

Engineer 81, 130

Woodworker 14, 34

Leather worker 9, 19

Textile worker 73, 132

Clothing worker 38, 76

Food industry 44, 69

Printer 11, 30

Construction industry 29, 47

Painters 7, 16

Labourer 10, 14

Transport worker 52, 95

Warehouse 5, 17

Clerical work 39, 56

Sales work 92, 166

Service industries 101, 198

Professional worker 18, 43

Armed Forces 83, 154

*Drug ingestion*

Amphetamines 11, 20

Antihistamine 7, 10

Contraception 6, 11

Antibiotics 19, 23

Analgesics 43, 78

Other antinausea drug 9, 9

Antacids 28, 49

Benzodiazepine 68, 110

Anticonvulsant 9, 16

Anti-inflammatory 55, 82

Bronchodilators 15, 31

Steroids 25, 51

Endocrine 9, 10

*Family illnesses*

Confirmed cancer in relations 56, 83

Infectious mononucleosis 12, 14

TB 31, 50

Rheumatoid arthritis 6, 19

Diabetes 34, 52

Asthma 40, 58

Eczema/dermatitis 11, 12

Psoriasis 4, 6

*Social characteristics*

Jews 5, 7

Sibship size[b]

Cigarette smoker 197, 292[c]

Wine drinker 27, 28

Spirit drinker 37, 77

Pet owner 263, 448

Foreign travel 140, 304

[a]Number of positive cases, control responses; [b]Various comparisons i.e. 0+1 *versus* 2+ and 0 *versus* 1+ and 0, 1, 2, 3 *versus* 3+ etc.; [c]*Versus* modified controls, i.e. the control group, eliminating those controls with smoking related conditions at time of interview.

*Skin diseases*

As shown in Table III no excess risk is associated with a previous history of eczema/dermatitis and the excess risks shown for other skin conditions are largely confined to the CLL group excluding MLL. The excess risk is due largely to past skin malignancies of several histopathological types and treatment by radiotherapy or other steroids increases the risk.

*Past medical conditions*

A history of past malignancy (excluding skin cancers) produced an overall twofold risk (RR = 2.69, $P = 0.002$) confined largely to the CLL group excluding MLL. No one solid tumour type accounted for this excess.

Table IV gives pooled results for other past medical conditions. There is a significant negative relationship with past appendectomies. With this exception all other associations show significant excesses particularly with various forms of past infection. These infections normally predate the diagnosis of CLL/MLL by many years. However, herpes zoster infections are more common within 2 years of CLL & MLL diagnosis, whilst its appearance more than 5 years before diagnosis is almost the same in as the control population. The herpes zoster infections are specifically linked with the CLL subgroup (i.e. excluding MLL).

A strong association was also observed with migraine almost exclusively in women ($P = 0.008$) and with heart disease especially hypertension and myocardial infarction in men ($P = 0.01$). However no excess of cigarette smoking was associated with any of the case subgroups (overall RR = 0.8, $P = 0.19$). Finally osteoarthritis in females with CLL excluding MLL proved a significant risk factor (RR = 2.1, $P = 0.04$), but not in males.

*Past therapy*

Table V summarizes some risks linked with previous therapy. The risks associated with past radiotherapy may be due to treatment for skin malignancies or internal solid tumours and would depend on the dose received by circulating lymphocytes. The association with drugs used to treat arterial conditions seems likely to be related to the excess of heart disease and hypertension already noted. Although a wide variety of drugs was involved, only digoxin showed significant associations ($P = 0.04$). Finally phenylbutazone showed a strong association ($P = 0.006$) when taken within 10 years of diagnosis of CLL/MLL for various arthritic conditions. No significant risk could be found for other anti-inflammatory drugs.

*Familial diseases*

The results for associated family illnesses are shown in Table VI. The association with other malignancies is confined to blood relations. Overall there is a weak familial excess due to a variety of lymphoid and myeloid malignancies. Although most risks are greater than unity no significant excess is achieved.

There is a clear excess of cases with a family history of multiple sclerosis (MS). This incorporates both spouses and first degree blood relatives. Unlike the link with leukaemia, this excess is not confined to blood relations: two spouse pairs were observed although the majority of this association is due to sib pairs.

*Social and occupational factors*

There were no significant excesses in social characteristics nor any occupational links except for the small absolute excess of electrical workers referred to previously.

### Discussion

The results of this study tend to support a multifactorial aetiology in the production of these malignancies and in general risks are common to both the haematogenous and solid lymphomatous forms of the disease.

The association found by Linos (1981) and Karchmer *et al.* (1974) between skin cancers and these conditions, not confined to the radiotherapy treated group, has been confirmed. However, no temporal link between skin cancer and CLL/MLL has been established and may be due to common aetiological factors rather than sequential steps in the leukaemic process. Skin repair mechanisms and other aspects of skin immunity may be important although this study has not revealed excesses of malignancy in groups who might be supposed to have excessive exposure to sunlight, such as farmers.

It might also be deduced from our results that systemic and skin immunity may be impaired from the association found with herpes zoster infection which was also reported in the tristate study (Gibson *et al.*, 1976) where, in addition, an association with 'rheumatism' and arthritis was noted. Unlike other work no link with rheumatoid arthritis was found. Of the other infections that show excesses in this study chronic bronchitis and chronic ear infection are novel

**Table III** Chronic lymphocytic leukaemia: Case control study: Association with past skin conditions

| *Eczema/dermatitis* | | *No. cases* | *No. controls* | *RR* | *95% confidence limit* | *2 tail P* |
|---|---|---|---|---|---|---|
| CLL | Male | 16 | 26 | 1.0 | 0.5–2.0 | 0.91 |
| | Female | 13 | 18 | 1.3 | 0.6–2.8 | 0.47 |
| MLL | Male | 5 | 11 | 0.7 | 0.2–2.3 | 0.61 |
| | Female | 5 | 7 | 1.1 | 0.3–3.9 | 0.84 |
| All other skin conditions[a] CLL | Male | 26 | 15 | 3.4 | 1.8–6.5 | <0.001 |
| | Female | 13 | 16 | 1.5 | 0.7–3.3 | 0.29 |
| MLL | Male | 4 | 8 | 0.8 | 0.2–2.9 | 0.77 |
| | Female | 6 | 6 | 1.6 | 0.5–5.5 | 0.42 |
| Basal cell carcinoma CLL | Male | 7 | 2 | 6.6 | 1.6–26.2 | 0.008 |
| | Female | 6 | 3 | 3.6 | 0.9–13.5 | 0.06 |
| Other skin cancers CLL | Male | 4 | 0 | a | | |
| | Female | 2 | 1 | a | | |
| Any skin cancer with radiotherapy CLL | Male and Female | 6 | 1 | 11.1 | 2.0–60.2 | 0.006 |
| Any skin cancer without radiotherapy CLL | Male and Female | 13 | 5 | 4.8 | 1.9–12.4 | 0.002 |

[a]Too few numbers for analysis; [b]Including skin cancer.

**Table IV** Chronic lymphocytic leukaemia: Case control study: Past medical history[a]

| *Condition* | *No. cases* | *No. controls* | *RR* | *95% confidence limits* | *2 tail P* |
|---|---|---|---|---|---|
| Appendectomy | 52 | 125 | 0.7 | 0.5–0.9 | 0.02 |
| Migraine | 10 | 4 | 4.4 | 2.0–12.7 | 0.008 |
| Scarlet fever | 11 | 6 | 3.2 | 1.2–8.3 | 0.02 |
| Herpes zoster | 36 | 34 | 1.9 | 1.2–3.1 | 0.01 |
| Diagnosed 0–1 years previously | 8 | 4 | 3.6 | 1.2–11.2 | 0.02 |
| 2–4 | 8 | 6 | 2.4 | 0.9–6.7 | 0.10 |
| 5+ | 17 | 23 | 1.3 | 0.7–2.5 | 0.39 |
| Chronic ear infection | 23 | 21 | 1.9 | 1.1–3.5 | 0.03 |
| Bronchitis | 21 | 17 | 2.2 | 1.2–4.1 | 0.02 |
| All heart disease includes: | 62 | 71 | 1.6 | 1.1–2.3 | 0.01 |
| Hypertension | 37 | 41 | 1.6 | 1.0–2.6 | 0.04 |
| Myocardial infarction | 15 | 13 | 2.0 | 1.0–4.2 | 0.05 |
| Past malignancy | 24 | 16 | 2.7 | 1.4–5.0 | 0.002 |

[a]Pooled sexes, pooled diagnosis.

**Table V** Chronic lymphocytic leukaemia: Case control study: Past medical treatment[a]

| *Condition* | *No. cases* | *No. controls* | *RR* | *95% confidence limit* | *2 tail P* |
|---|---|---|---|---|---|
| Past radiotherapy | 16 | 10 | 2.8 | 1.3–6.1 | 0.01 |
| Antihypertensives and diuretics and related drugs | 91 | 114 | 1.5 | 1.1–2.1 | 0.01 |
| Phenylbutazone within 10 years of diagnosis | 27 | 21 | 2.2 | 1.3–4.0 | 0.006 |

[a]Pooled sexes, pooled diagnosis.

**Table VI** Chronic lymphocytic leukaemia: Case control study: Family history[a]

| *Condition* | *No. cases* | *No. controls* | *RR* | *95% confidence limit* | *2 tail P* |
|---|---|---|---|---|---|
| Lymphoma or leukaemia in families | 20 | 20 | 1.8 | 0.9–3.3 | 0.08 |
| CONFIRMED CASES: | | | | | |
| NHL in families | 4 | 2 | 3.4 | 0.7–17.1 | 0.13 |
| HD in families | 3 | 7 | 0.7 | 0.2–2.8 | 0.64 |
| Lymphoid leukaemia in families | 5 | 2 | 4.3 | 0.9–19.5 | 0.13 |
| Myeloid leukaemia in families | 4 | 2 | 3.4 | 0.7–17.1 | 0.77 |
| 'Other' leukaemia in families | 5 | 7 | 1.2 | 0.4–3.9 | 0.73 |
| Multiple sclerosis in families | 15 | 11 | 2.4 | 1.1–5.2 | 0.03 |

[a]Pooled sexes, pooled diagnosis.

observations, whilst scarlet fever (often many years ago) was previously observed in the pilot study (Bernard *et al.*, 1984). An interesting possible new link with migraine was observed but other conditions, such as asthma or eczema, were not shown to be in excess contrary to our pilot study results (Bernard *et al.*, 1984) and one report from elsewhere showing an association with eczema (Gibson *et al.*, 1976).

The association between heart disease and treatment with related drugs is new and unexpected. It was asserted many years ago that phenylbutazone may be linked with leukaemia induction due to its noxious side-effects but when this was critically addressed by a case control study by Friedman (1982) he showed a link with prior musculoskeletal diseases rather than treatment. In our study phenylbutazone had mainly been prescribed for arthritic conditions occurring some time prior to diagnosis and usually described as osteo-arthritis.

The familial links with CLL have been reported before (Gunz *et al.*, 1975; Conley *et al.*, 1980). In this survey the most common relationship is in sib pairs. No excess of cases in Jewish patients was observed although this was found in the pilot study (Bernard *et al.*, 1984) along with other lymphomas and has been described in other reports (Bartal *et al.*, 1978).

The possible link with MS has been reported elsewhere (Bernard *et al.*, 1986) and might be relevant to the observation by Koprowski *et al.* (1985) who have claimed to find HTLV-like sequences in spinal fluid leucocytes from sufferers of MS. Broad parallels can also be drawn with MS where cases appear to have increased numbers of prior infections notably acquired at older ages (Phadke & Downie, 1987).

The view that these malignancies arise because of genetic susceptibility associated with some form of immune perturbation and infective disorder is supported by the following observations in this study: Genetic susceptibility is particularly linked to the increased incidence of the malignancies observed in families. The association with immune perturbation is supported by the occurrence of excess prior skin and other internal malignancies, possibly indicating a lowering of normal immune surveillance and the excess of chronic and severe infections. Finally the link with infectious agents is supported by the relation with MS and a variety of infections.

This study could not have been accomplished without the assistance of numerous colleagues within the Yorkshire Health Region who have allowed us access to their patients. The work was funded by the Leukaemia Research Fund and we would specifically like to acknowledge the assistance of Mary Brown, Jill Collins, Sylvia Craven, Fiona Landells, Helen Lilley, Felicity Ludolf, Anne Mainwaring, Jan Parker, Jane O'Sullivan, Bernice Pearlman, Carole Startin and Brenda Waller who have worked for several years with us on this project. The support of the Yorkshire Cancer Research Campaign and the Yorkshire Regional Cancer Registry is also gratefully acknowledged.

### References

BARTAL, A., BENTWICH, Z., MANNY, N. & IZAK, G. (1978). Ethnical and Clinical aspects of chronic lymphocytic leukaemia in Israel. *Acta Haematol.*, **60,** 161.

BERNARD, S.M., CARTWRIGHT, R.A., BIRD, C.C., RICHARDS, I.D.G., LAUDER, I. & ROBERTS, B.E. (1984). Aetiologic factors in lymphoid malignancies: A case-control epidemiological study. *Leukemia Res.*, **8,** 681.

BERNARD, S.M., CARTWRIGHT, R.A., DARWIN, C.M. & 4 others (1987). Hodgkin's disease: Case control epidemiological study in Yorkshire. *Br. J. Cancer*, **55,** 85.

CONLEY, C.L., MISITI, J. & LASTER, A.J. (1980). Genetic factors pred-disposing to chronic lymphocytic leukaemia and to auto-immune disease. *Medicine*, **59,** 323.

FRIEDMAN, G.D. (1982). Phenylbutazone, musculoskeletal disease and leukaemia. *J. Chron. Dis.*, **34,** 233.

GIBSON, R., GRAHAM, S., LILIENFELD, A., SCHUMANN, L., LEVIN, M. & SWANSON, M. (1976). Epidemiology of diseases in adult males with leukaemia. *J. Natl Cancer Inst.*, **56,** 891.

GUNZ, F.W., GUNZ, J.P., VEALE, A.M.O., CHAPMAN, C.J. & HOUSTON, I.B. (1975). Familial leukaemia: A study of 909 families. *Scand. J. Haematol.*, **15,** 117.

KARCHMER, R.K., MELLMAN, J.A., CALDWELL, G.G. & CHIN, T.D.Y. (1974). Previous and simultaneous cancers in patients with leukaemia. *J. Chronic Dis.*, **27,** 5.

KOPROWSKI, H., DE FRIETAS, E.C., HARPER, M.E. & 5 others (1985). Multiple sclerosis and human T-cell lymphotropic retroviruses. *Nature*, **318,** 154.

LINOS, A. (1981). Leukaemia and prior malignant and haematologic disease: A case-control study. *Am. J. Epidemiol.*, **113,** 285.

PHADKE, J.G. & DOWNIE, A.W. (1987). Epidemiology of multiple sclerosis in the north-east (Grampian Region) of Scotland – an update. *J. Epidemiol. Comm. Health*, **41,** 5.

ROTHMAN, K. & BOICE, J.D. (1982). Epidemiologic analysis with a programmable calculator. *NIH publication no.* 79–1649.